# Immunogenetic modulation of endothelial inflammation by the LIPG −384A/C promoter variant influences COVID-19 severity

**DOI:** 10.3389/fimmu.2026.1785738

**Published:** 2026-06-03

**Authors:** HariOm Singh, Aishwarya Nair, Meenakshi Bhattacharya, Gaurav Tripathi, Shamama Nishat, Abdullah F. AlAsmari, Nemat Ali

**Affiliations:** 1 BSL-4, ICMR–National Institute of Virology, Pune, India; 2 Department of Medicine, Government Medical College & Hospital, Aurangabad, India; 3 Temple University Hospital, Philadelphia, PA, United States; 4 Comprehensive Cancer Centre, Wexner Medical Centre, Ohio State University, Columbus, OH, United States; 5 Department of Pharmacology and Toxicology, College of Pharmacy, King Saud University, Riyadh, Saudi Arabia

**Keywords:** COVID-19, EL-384A/C polymorphism, endothelial lipase, genetic susceptibility, hyperferritinemia, mild COVID-19

## Abstract

**Background:**

Severe COVID-19 is characterized by dysregulated host immune responses and immune-mediated endothelial injury, culminating in microvascular dysfunction and systemic inflammation. Endothelial lipase (EL), encoded by the *LIPG* gene, regulates endothelial lipid metabolism and inflammatory signaling pathways that influence immune–endothelial interactions. Despite its relevance to vascular immunopathology, the immunogenetic contribution of *LIPG* promoter variants to immune-driven endothelial dysfunction in COVID-19 remains poorly defined.

**Methods:**

We performed a case–control study including 151 RT-PCR–confirmed COVID-19 patients stratified according to disease severity and 152 healthy controls. Genotyping of the *LIPG* −384A/C (rs3813082) promoter variant was carried out using polymerase chain reaction–based allelic discrimination. Associations between genotype and disease severity were evaluated alongside inflammatory and immune-related parameters, including serum ferritin and total leukocyte count. *In silico* analyses were conducted to assess the impact of the promoter variant on transcription factor binding sites involved in immune and inflammatory regulation.

**Results:**

The *LIPG* −384C allele was significantly associated with an increased risk of severe COVID-19 and a heightened inflammatory profile. Individuals carrying the risk allele demonstrated significantly elevated ferritin levels and leukocyte counts, indicative of amplified systemic immune activation. Computational functional annotation revealed that the −384A/C substitution modifies transcription factor binding motifs linked to inflammatory and immune-responsive pathways, supporting a mechanistic role for this variant in regulating endothelial immune signaling.

**Conclusion:**

This study identifies the *LIPG* −384A/C promoter variant as a novel immunogenetic determinant of COVID-19 severity, potentially mediating disease progression through modulation of immune-driven endothelial inflammation. Our findings underscore the critical role of host immunogenetic factors at the immune–endothelial interface in shaping inflammatory responses and clinical outcomes in COVID-19, providing insight into vascular immunopathology and host susceptibility.

## 1 Introduction

Coronavirus disease 2019 (COVID-19), caused by severe acute respiratory syndrome coronavirus 2 (SARS-CoV-2), manifests with a broad clinical spectrum ranging from asymptomatic infection to severe pneumonia, multiorgan failure, and death (1, 2). Although the majority of individuals experience mild illness, a substantial proportion progress to severe disease, with host factors such as age, comorbidities, and genetic background contributing to inter-individual variability in clinical outcomes (3–8).

A defining feature of severe COVID-19 is dysregulated inflammation accompanied by endothelial injury, microvascular thrombosis, and acute respiratory distress syndrome (9, 10). Mounting evidence indicates that COVID-19 is fundamentally an endothelial and vascular disease (11–13). Autopsy and clinical studies demonstrate diffuse endothelial activation, endotheliitis, disruption of the endothelial glycocalyx, hypercoagulability, and widespread microvascular thrombosis (11–17). Endothelial cells regulate vascular tone, inflammatory signaling, and barrier integrity; their dysfunction contributes not only to acute disease severity but also to long-term post-acute sequelae (18, 19). Mechanistic studies further show that SARS-CoV-2 induces oxidative stress, leukocyte adhesion, endothelial senescence, endothelial-to-mesenchymal transition, and pronounced vascular inflammation (20).

Endothelial lipase (EL), encoded by the *LIPG* gene, is an endothelial-derived phospholipase that modulates HDL metabolism and vascular inflammatory responses. EL expression increases during inflammatory and endothelial stress, making it a biologically plausible mediator in COVID-19–related endothelial dysfunction (21). EL shares 44% sequence homology with lipoprotein lipase and 41% with hepatic lipase and exhibits higher phospholipase than triglyceride lipase activity (22–24). Genetic studies indicate that EL variants influence HDL-C levels and cardiovascular risk (25–27). Among these, the promoter polymorphism EL-384A/C (rs3813082) alters transcriptional regulation and has been associated with lipid traits across multiple populations (28–32).

Genetic variants located within promoter regions may influence transcription factor binding and gene expression, thereby modifying endothelial inflammatory responses (33–35). The EL-384A/C (rs3813082) polymorphism lies within a regulatory region of the *LIPG* promoter and has been predicted to affect binding sites for transcription factors involved in inflammatory signaling pathways, including NF-κB, AP-1, SP1, and GATA. These regulatory factors play critical roles in cytokine production, endothelial activation, and immune cell recruitment during viral infections. Therefore, variation at this locus may influence endothelial inflammatory responses and contribute to inter-individual variability in host responses to SARS-CoV-2 infection.

Despite the central role of endothelial biology in COVID-19, no study to date has examined whether the EL-384A/C promoter variant influences COVID-19 susceptibility, disease severity, or inflammatory biomarker profiles. Given the importance of endothelial activation and HDL-related pathways in SARS-CoV-2 pathophysiology, EL genetic variation may modulate host responses to infection.

Therefore, the present study investigated the association of the EL-384A/C polymorphism with COVID-19 susceptibility, disease severity, and biochemical markers, complemented by *in silico* functional analysis to elucidate its potential regulatory role in endothelial inflammation.

## 2 Materials and methods

### 2.1 Sample size determination

Sample size was calculated based on the reported prevalence of severe COVID-19 (37%) (3) and the EL-384C allele frequency in healthy individuals (14.1%) (32). Using these parameters, a minimum of 151 participants was required to achieve 80% power at α = 0.05 for detecting a significant genetic association. The final study included 151 COVID-19 cases and 152 healthy controls.

### 2.2 Study design and participants

This hospital-based case–control study was conducted from November 2021 to February 2023. A total of 151 RT-PCR–confirmed COVID-19 patients and 152 age- and sex-matched healthy controls (SARS-CoV-2 RT-PCR negative) were enrolled from Government Medical College, Aurangabad.

The study specifically investigated the association of the EL-384A/C (rs3813082) promoter polymorphism with COVID-19 susceptibility, disease severity, and biochemical markers.

### 2.3 Inclusion and exclusion criteria

Disease severity was classified according to standard clinical, laboratory, and radiological criteria. Inclusion and exclusion criteria for all groups are summarized in **Table 1**.

**Table 1:** Inclusion and exclusion criteria for study participants.

Group	Inclusion criteria	Exclusion criteria
Critical cases	RT-PCR–confirmed COVID-19 with any of: PaO_2_/FiO_2_ ≤200 mmHg; shock requiring vasopressors (MAP ≥65 mmHg); septic shock; multiorgan dysfunction; respiratory failure requiring mechanical ventilation.	TB or Hepatitis B/C coinfection, chronic alcohol or drug abuse
Severe cases	RT-PCR–confirmed COVID-19 with: respiratory rate ≥30/min, SpO_2_ ≤93% at rest, or PaO_2_/FiO_2_ ≤300 mmHg.	Same as above
Moderate cases	PaO_2_/FiO_2_ 200–300; respiratory rate >24/min; SpO_2_ <93% on room air.	Same as above
Mild cases	Fever, fatigue, sore throat, anosmia, and no radiological pneumonia.	Same as above
Asymptomatic cases	RT-PCR–positive with no symptoms.	Comorbidities (diabetes, hypertension, cardiac/respiratory disease), TB, Hepatitis B/C, alcohol or drug use
Healthy controls	Age- and sex-matched; RT-PCR–negative for SARS-CoV-2.	Same as above

To minimize potential clinical confounding, individuals with major comorbid conditions known to influence COVID-19 severity—including diabetes mellitus, hypertension, cardiovascular disease, chronic respiratory disorders, and other severe systemic illnesses—were excluded from the study cohort wherever possible during recruitment. This approach was adopted to better evaluate the independent association between the LIPG −384A/C (rs3813082) polymorphism and disease severity.

### 2.4 Ethical approval

The study was approved by the Institutional Ethics Committees of Government Medical.

College, Aurangabad, and ICMR–NARI, Pune (Approvals: NARI/EC/Approval/20-21;

Pharmac/IEC-GMCA/IEC-Approval/008-2021). Written informed consent was obtained from all participants. All procedures adhered to the Declaration of Helsinki.

### 2.5 DNA extraction and genotyping

Peripheral blood (2 mL) was collected in EDTA tubes and stored at −70 °C until analysis.

Genomic DNA was extracted using the Qiagen DNA Isolation Kit.

Genotyping of EL-384A/C (rs3813082) was performed using PCR–Restriction Fragment.

Length Polymorphism (PCR-RFLP) according to published primers (Kobayashi et al., 2003).

Primer sequences and restriction details are provided in **Table 1.1**.

**Table 1.1:** PCR–RFLP details for EL (−384A/C) polymorphism.

SNP	Primer sequence (5′–3′)	Enzyme	Product (bp)	Fragment pattern
EL (−384A/C)	F: 5′-TAGCTCCGCCGGGTTATTGTGC-3′ R: 5′-CCAGATCCTCCTCTCCCCACTG-3′	HhaI	254 bp	AA: 254 bp AC: 254 bp + 142 bp + 112 bp CC: 142 bp+ + 112 bp

PCR amplification was performed with an initial denaturation at 95 °C for 5 minutes, followed by 35 cycles of denaturation at 95 °C for 1 minute, annealing at 58 °C for 30 seconds, and extension at 72 °C for 1 minute, with a final extension at 72 °C for 7 minutes.

PCR products and digested fragments were visualized on 2–3% agarose gels stained with ethidium bromide.

### 2.6 Quality control

To ensure genotyping accuracy, 20% of samples were randomly selected for repeat genotyping, and 10% of samples were further validated by Sanger sequencing.

### 2.7 In silico functional analysis

Functional consequences of the EL-384A/C promoter variant were evaluated using multiple regulatory genomics tools:

Analyses were conducted using GRCh38/hg38 coordinates. Significant changes in TF binding scores or eQTL associations (p ≤ 0.05) were considered potentially functional (**Table 1.2**).

**Table 1.2:** Bioinformatic tools and databases used for functional annotation and in silico analysis of the LIPG rs2000813 variant and its potential regulatory role in endothelial inflammation and lipid metabolism.

Objective	Tool/database	Analysis
Regulatory annotation	Ensembl, UCSC, RegulomeDB	Chromatin marks (H3K4me3, H3K27ac), DNase hypersensitivity
TF binding prediction	JASPAR, PROMO	Gain or loss of transcription factor motifs
Expression/eQTL	GTEx	Effect of C-allele on LIPG expression in blood and arterial tissues
Conservation	PhyloP, PhastCons	Evolutionary conservation assessment
Pathway mapping	STRING, GeneMANIA	Interaction networks involving EL in endothelial inflammation and lipid metabolism

### 2.8 Statistical analysis

All statistical analyses were performed using IBM SPSS Statistics (IBM Corp., Armonk, NY, USA). Continuous variables are presented as mean ± standard deviation (SD), while categorical variables are expressed as frequencies and percentages. The Hardy–Weinberg equilibrium (HWE) for genotype distribution in the control group was assessed using the χ² test. Differences in genotype and allele frequencies between COVID-19 patients and healthy controls were evaluated using the χ² test or Fisher’s exact test, where appropriate.

To examine the relationship between the EL-384A/C (rs3813082) polymorphism and COVID-19 outcomes, multivariable logistic regression analysis was performed to estimate odds ratios (ORs) and 95% confidence intervals (CIs) for associations with COVID-19 susceptibility, disease severity, and biochemical parameters, including CRP, D-dimer, ferritin, SGOT, bilirubin, hemoglobin, creatinine, platelet count, blood urea, and total leukocyte count (TLC). The models were adjusted for age and sex, as these represent the most consistently reported demographic determinants of COVID-19 severity.

Genetic associations were evaluated under multiple inheritance models, including allelic (C vs A), dominant (AC + CC vs AA), recessive (CC vs AC + AA), and additive (per-C allele) models using logistic regression. Model fit was evaluated using the Akaike Information Criterion (AIC), and the additive model was considered the primary model unless otherwise specified.

Trends in disease severity across genotype categories were assessed using the Cochran–Armitage trend test. To control for potential type I errors arising from multiple comparisons, Benjamini–Hochberg false discovery rate correction was applied. In addition, Receiver Operating Characteristic curve (ROC) analysis was performed to evaluate the discriminatory performance of selected biochemical markers for predicting elevated ferritin and TLC levels. A two-tailed P value < 0.05 was considered statistically significant.

## 3 Results

### 3.1 Demographic and clinical characteristics of the study population

A total of 151 RT-PCR–confirmed COVID-19 patients and 152 healthy controls from Western India were enrolled. The mean age of patients was 50.9 ± 6.3 years, comparable to controls (48.3 ± 4.5 years), with no significant age difference (**Table 2**).

**Table 2:** Demographic and clinical characteristics of study participants.

Parameter	COVID-19 patients (n = 151)	Healthy controls (n = 152)
Age (years, mean ± SD)	50.92 ± 6.34	48.31 ± 4.53
Sex (Male/Female)	96 (63.6%)/55 (36.4%)	67 (44.1%)/85 (55.9%)
Ethnicity	Western India	Western India
Disease severity (CT score)	Critical: 11 (7.3%) Severe: 43 (28.5%) Moderate: 63 (41.7%) Mild: 34 (22.5%)	—
Elevated CRP	53 (35.1%)	—
Elevated Ferritin	68 (45.0%)	—
Elevated TLC	43 (28.5%)	—
Elevated D-dimer	7 (4.6%)	—

Values are presented as n (%) unless otherwise stated.

Among patients, 63.6% were male and 36.4% female, whereas controls included 44.1% males and 55.9% females. Based on CT severity scores, patients were classified as critical (7.3%), severe (28.5%), moderate (41.7%), and mild (22.5%). Elevated inflammatory and hematological markers were observed in subsets of patients, including CRP (35.1%), ferritin (45.0%), TLC (28.5%), and D-dimer (4.6%) (**Table 2**).

### 3.2 Sex-specific distribution of EL-384A/C (LIPG rs3813082) in COVID-19 patients

Sex-stratified analysis revealed significant differences in genotype and allele distributions among COVID-19 patients (**Table 3**). The AC genotype was more frequent in female patients than males (50.9% vs. 31.2%; P = 0.02; OR = 0.44, 95% CI: 0.21–0.92).

**Table 3:** Combined genotype and allele distribution of EL-384A/C (LIPG rs3813082) in COVID-19 patients by sex.

Category	Variant	Male COVID-19 (n = 96/alleles = 192)	Female COVID-19 (n = 55/alleles = 110)	P-value	OR (95% CI)
Genotype	AA	66 (68.8%)	27 (49.1%)	Reference	1.00
	AC	30 (31.2%)	28 (50.9%)	**0.02**	**0.44 (0.21–0.92)**
Allele	A	162 (84.37%)	82 (74.54%)	Reference	1.00
	C	30 (15.62%)	28 (25.45%)	**0.05**	**0.54 (0.29–1.01)**

N, total number of subjects; (%), frequency of genotypes/alleles. Odds ratios (OR) were estimated using logistic regression under an additive genetic model. Genotype frequencies were compared using χ² tests. The AA genotype and A allele were used as reference categories for the EL-384A/C polymorphism. Significant values (P ≤ 0.05) are shown in bold.

Similarly, the C allele frequency was higher in females (25.5%) than males (15.6%; P = 0.05; OR = 0.54, 95% CI: 0.29–1.01), suggesting a sex-dependent enrichment of the protective C allele in females.

### 3.3 Association of EL-384A/C with COVID-19 susceptibility

Genotype and allele frequencies were comparable between patients and controls (**Table 4**). The AA genotype was observed in 61.6% of cases and 65.8% of controls, while the AC genotype was slightly more frequent in cases (38.4% vs. 29.6%); however, this difference was not statistically significant (P = 0.22; OR = 1.39, 95% CI: 0.83–2.31).

**Table 4:** Combined genotype and allele distribution of EL-384A/C in COVID-19 patients and healthy controls (susceptibility analysis).

Category	Variant	COVID-19 (n = 151/alleles = 302)	Controls (n = 152/alleles = 304)	P-value	OR (95% CI)
Genotype	AA	93 (61.6%)	100 (65.8%)	Reference	1.00
	AC	58 (38.4%)	45 (29.6%)	0.22	1.39 (0.83–2.31)
	CC	0 (0.0%)	7 (4.6%)	—	—
Allele	A	244 (80.79%)	245 (80.59%)	Reference	1.00
	C	58 (19.20%)	59 (19.40%)	0.96	0.99 (0.65–1.51)

N, total number of subjects; (%), frequency of genotypes or alleles. Odds ratios (OR) were estimated using logistic regression under genotype and allelic models, adjusted for age and sex. Genotype frequencies were compared using the χ² test, and allele frequencies were evaluated using allelic association analysis. The AA genotype and A allele were used as reference categories for the EL-384A/C polymorphism. Hardy–Weinberg equilibrium was assessed in the control group using the χ² test (P > 0.05 indicates equilibrium).

**Table 4A:** Dominant and recessive model analysis of EL-384A/C.

Genetic model	Comparison	OR (95% CI)	P value
Dominant	AC+CC vs AA	1.34 (0.81–2.22)	0.25
Recessive	CC vs AC+AA	0.19 (0.02–1.56)	0.12

Genetic association was evaluated using dominant and recessive inheritance models by logistic regression adjusted for age and sex.

Allelic analysis similarly showed no significant difference in C-allele frequency between cases(19.2%) and controls (19.4%; P = 0.96). Genotype distributions in the control groupconformed to Hardy–Weinberg equilibrium (P = 0.50), confirming the reliability of the genotyping results (**Supplementary Table 3**).

To further evaluate the potential association of the EL-384A/C polymorphism with COVID-19 susceptibility, additional analyses were performed under dominant (AC + CC vs AA) and recessive (CC vs AC + AA) genetic models. Under both models, no statistically significant association was observed (**Table 4A**), consistent with the results of the genotype and allelic analyses.

### 3.4 Association of EL-384A/C with COVID-19 disease severity

Stage-specific analysis demonstrated enrichment of the C allele in mild COVID-19 (50.0%) compared with moderate (18.3%), severe (2.3%), and critical disease (0%) (**Table 5**). Compared with severe/critical disease, the C allele was strongly associated with mild disease (OR = 4.15; P < 0.001).

**Table 5:** Association of EL-384A/C with COVID-19 disease severity: stage-specific allelic analysis.

Severity Stage	A allele n (%)	C allele n (%)	OR (95% CI)	P-value
Critical	22 (100%)	0 (0.0%)	Reference	—
Severe	84 (97.7%)	2 (2.3%)	0.10 (0.01–0.78)	0.02
Moderate	103 (81.7%)	23 (18.3%)	0.94 (0.45–1.97)	0.92
Mild	34 (50.0%)	34 (50.0%)	**4.15 (1.90–9.08)**	**<0.001**

Allele frequencies are presented as n (%). Odds ratios (OR) and 95% confidence intervals (CI) were estimated using logistic regression under an allelic genetic model (C vs A), adjusted for age and sex, using the severe/critical group as the reference category. Trend analysis across ordered severity categories was further evaluated using the Cochran–Armitage test. P-values were derived from logistic regression or χ² tests where appropriate. Significant associations (P ≤ 0.05) are shown in bold.

**Figure 1:**
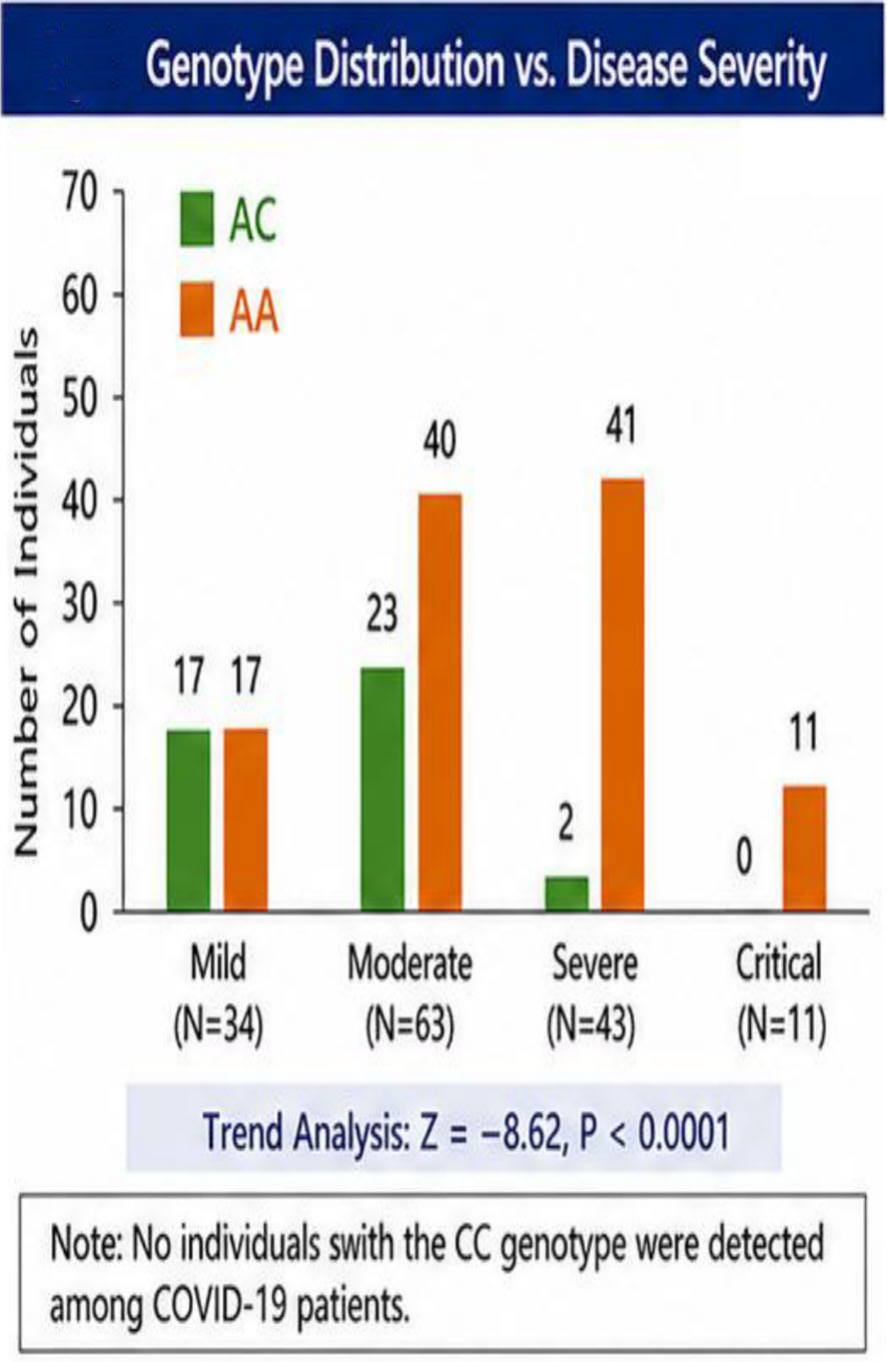
Distribution of EL-384A/C (rs3813082) genotypes across COVID-19 severity categories. The frequency of AA and AC genotypes is shown for mild, moderate, severe, and critical COVID-19 cases. N represents the number of individuals in each severity category (total n = 151). A progressive reduction of the AC genotype is observed with increasing disease severity, suggesting a protective association of the C allele.

Genotype distribution across severity categories is illustrated in **Figure 1**, showing a progressive loss of the AC genotype with increasing disease severity.

### 3.5 Severity trend and binary association analyses

Cochran–Armitage trend test revealed a highly significant inverse relationship between the EL-384A/C variant and COVID-19 severity (**Table 6**):

**Table 6:** Trend and binary association analyses of EL-384A/C with COVID-19 severity.

Analysis	Test	Statistic	P-value	Interpretation
Genotype trend (AC vs AA)	Cochran–Armitage	Z = −8.62	**<0.0001**	AC genotype decreases with severity
Allele trend (C vs A)	Cochran–Armitage	Z = −7.35	**2.01 × 10⁻¹³**	C allele enriched in mild disease
Binary allelic comparison	Fisher’s exact	OR = 22.05 (5.26–92.39)	**2.03 × 10⁻^10^**	Strong protection in non-severe cases

Cochran–Armitage trend tests were applied to evaluate ordered disease severity categories.Binary comparisons were assessed using Fisher’s exact test and χ² test.Multivariable logistic regression adjusted for age and sex is provided in **Supplementary Table 4**. Odds ratios (OR) with 95% confidence intervals (CI) indicate enrichment of the C allele in non-severe disease. Significant results are shown in bold.

- Genotype trend (AC vs. AA): Z = −8.62, P < 0.0001
- Allele trend (C vs. A): Z = −7.35, P = 2.01 × 10⁻¹³

**Figure 2:**
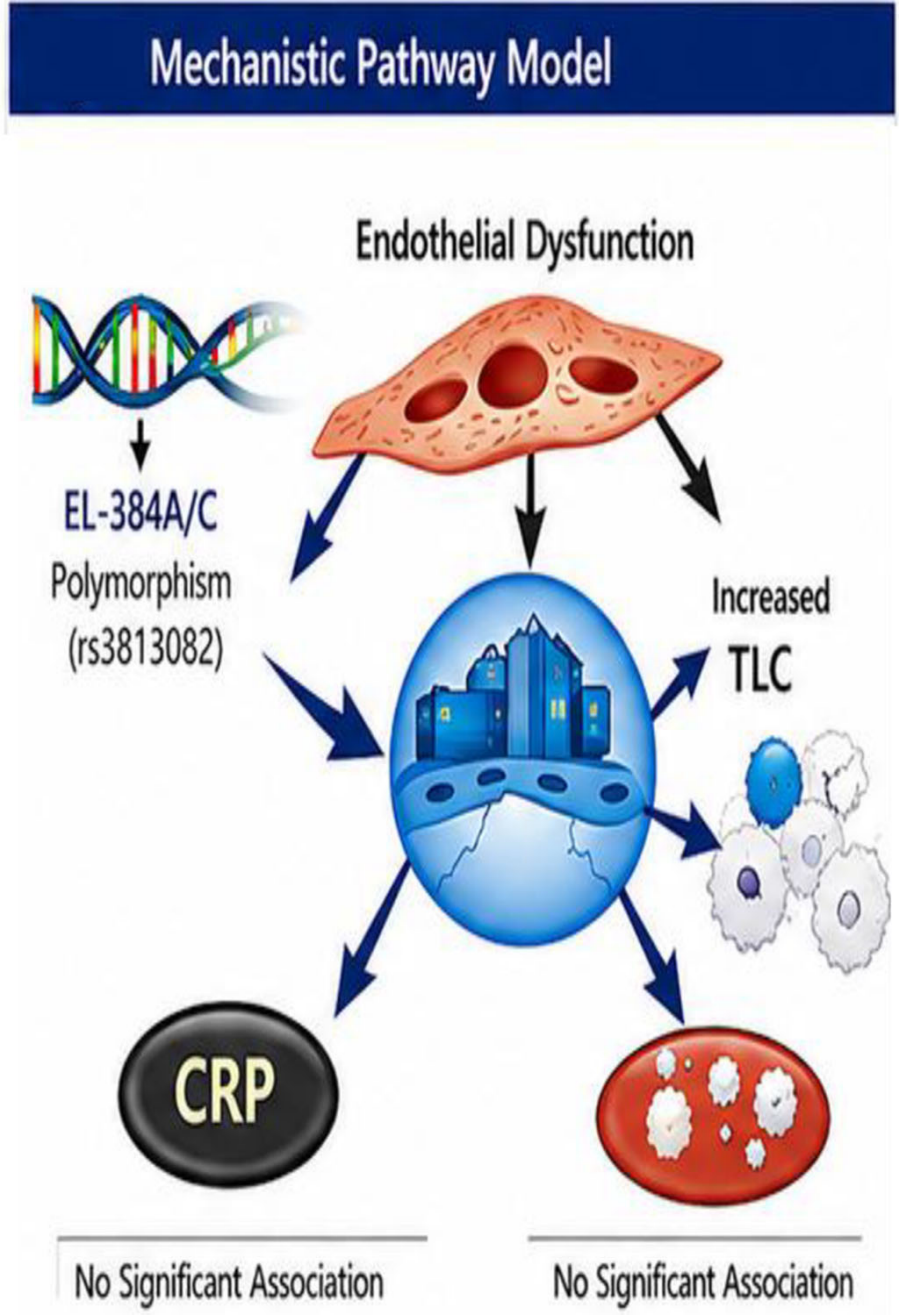
Hypothesized mechanistic model illustrating how the EL-384A/C promoter variant may influence endothelial inflammation and COVID-19 severity. The model integrates genetic association findings from the present study with in-silico predictions of altered transcription factor binding (NF-κB, SP1, and AP-1) and previously reported endothelial inflammatory signaling pathways.

In binary analysis (non-severe vs. severe/critical), the C allele was enriched in non-severe cases (OR = 22.05; 95% CI: 5.26–92.39; P = 2.03 × 10⁻^10^). These results demonstrate a robust protective effect of the C allele against disease progression. Based on the observed genetic associations and supported by in-silico regulatory predictions, a hypothetical mechanistic model illustrating how the EL-384A/C promoter variant may influence endothelial inflammatory responses is presented in **Figure 2**. The protective association remained significant after adjustment for age and sex inmultivariable logistic regression (**Supplementary Table 4**).

### 3.6 Association of EL-384A/C with inflammatory and hematological biomarkers

Key biomarker associations are summarized in **Table 7**:

**Table 7:** Key inflammatory biomarker associations with EL-384A/C.

Biomarker	Comparison	Variant	OR (95% CI)	P-value
Ferritin	Elevated vs Normal	AC vs AA	**5.89 (2.86–12.12)**	**<0.001**
		C vs A	**3.35 (1.72–6.57)**	**<0.001**
TLC	Elevated vs Normal	AC vs AA	**2.74 (1.33–5.68)**	**0.009**
CRP	Elevated vs Normal	AC vs AA	0.50 (0.23–1.03)	0.07
D-dimer	Elevated vs Normal	AC vs AA	2.22 (0.40–13.10)	0.42

Elevated biomarker levels were defined according to institutional clinical cut-offs. Odds ratios (ORs) were estimated using logistic regression under genotype (AC vs AA) and allelic (C vs A) models, adjusted for age and sex. P-values ≤ 0.05 are shown in bold.

- Ferritin: AC genotype strongly associated with hyperferritinemia (OR = 5.89; P < 0.001), with a parallel allelic effect (C vs. A: OR = 3.35; P < 0.001).
- TLC: AC genotype associated with elevated TLC (OR = 2.74; P = 0.009).
- CRP and D-dimer: No significant associations, although CRP showed a non-significant protective trend.

No meaningful associations were observed for creatinine, SGOT, bilirubin, platelet count,hemoglobin, or blood urea (**Supplementary Tables 1**, **2**).

### 3.7 Multiple-testing–corrected association summary

After Benjamini–Hochberg FDR correction, only disease severity, ferritin, and TLC remained significant (**Table 8**). Susceptibility and most biochemical markers lost significance, highlighting selectiveinflammatory relevance of the EL-384A/C variant. Multivariable-adjusted models yielded effect sizessimilar to unadjusted analyses (**Supplementary Table 4**).

**Table 8:** Summary of genetic associations after BH-FDR correction.

Outcome	Comparison	OR (95% CI)	Raw P	FDR-q	Interpretation
Susceptibility	AC vs AA	1.39 (0.86–2.24)	0.22	0.38	Not significant
Severity trend	C allele	—	2.01 × 10>⁻¹³	1.9 × 10>⁻¹²	Protective
Ferritin	AC vs AA	**5.90 (2.87–12.16)**	**7.5 × 10>⁻^7^**	**3.6 × 10>⁻^6^**	Significant
TLC	AC vs AA	**2.75 (1.33–5.68)**	**0.009**	**0.028**	Significant

False discovery rate (FDR) correction was applied using the Benjamini–Hochberg method. Associations with q ≤ 0.05 are shown in bold.

### 3.8 In-silico functional annotation of rs3813082

**Figure 3:**
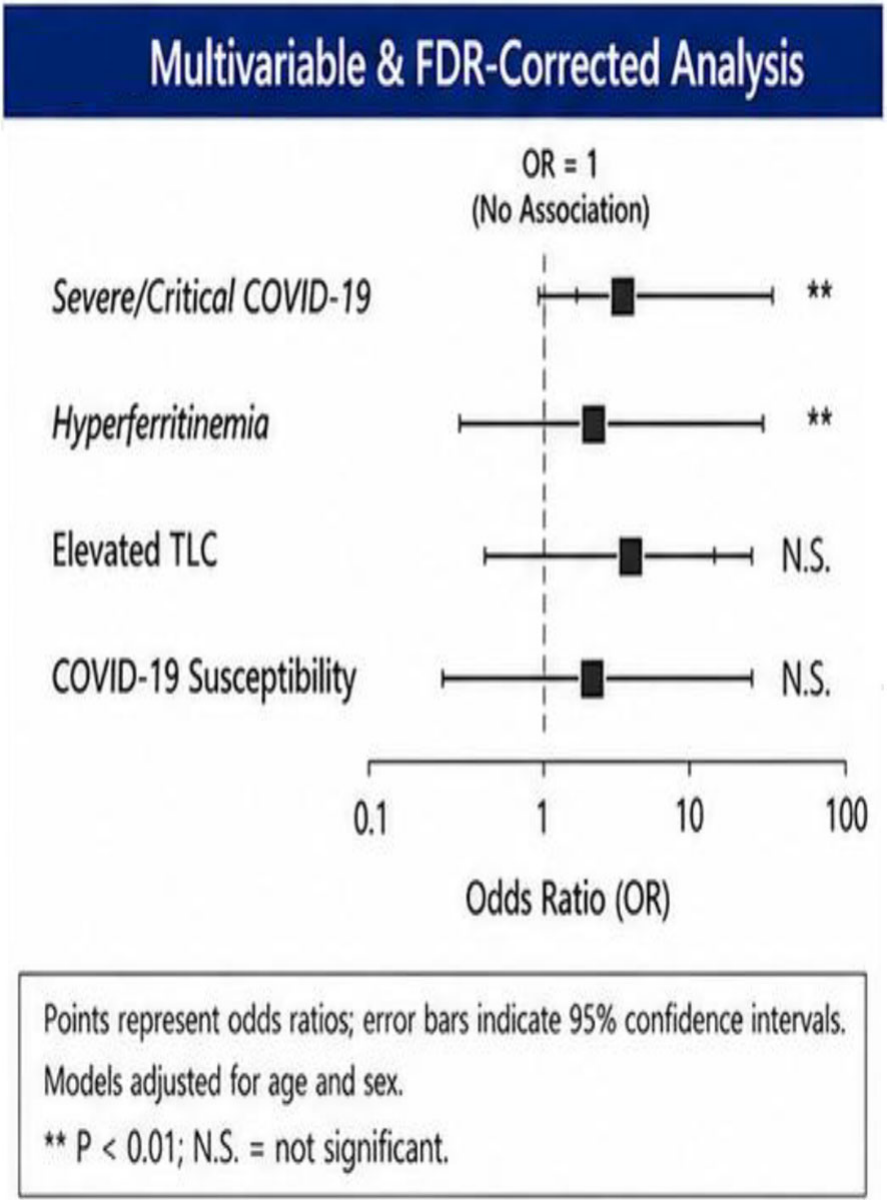
Multivariable and FDR-corrected analysis of the association between EL-384A/C (rs3813082) polymorphism and COVID-19 outcomes. Forest plot showing odds ratios (ORs) with 95% confidence intervals (CIs) for severe/critical COVID-19, hyperferritinemia, elevated total leukocyte count (TLC), and COVID-19 susceptibility. ORs were estimated using logistic regression under the genotype model (AC vs AA), adjusted for age and sex. Points represent OR estimates, and horizontal error bars indicate 95% CIs. The vertical dashed line indicates OR = 1 (no association). OR > 1 indicates increased risk, whereas OR < 1 indicates a protective effect. Statistical significance after Benjamini–Hochberg false discovery rate (FDR) correction is indicated (**), while “N.S.” denotes non-significant associations.

In-silico analyses indicated that rs3813082 resides within a regulatory promoter region of LIPG.RegulomeDB assigned a score of 2b, suggesting transcription factor binding. HaploReg andJASPAR/PROMO analyses predicted allele-specific modulation of NF-κB, SP1, and AP-1 motifs. GTEx data indicated an eQTL effect on LIPG expression (**Supplementary Table 7**). A mechanistic model linking EL-384A/C–dependent transcriptional regulation to endothelial inflammation is shown in **Figure 3**.

### 3.9 ROC curve analysis for prediction of elevated TLC

**Figure 4:**
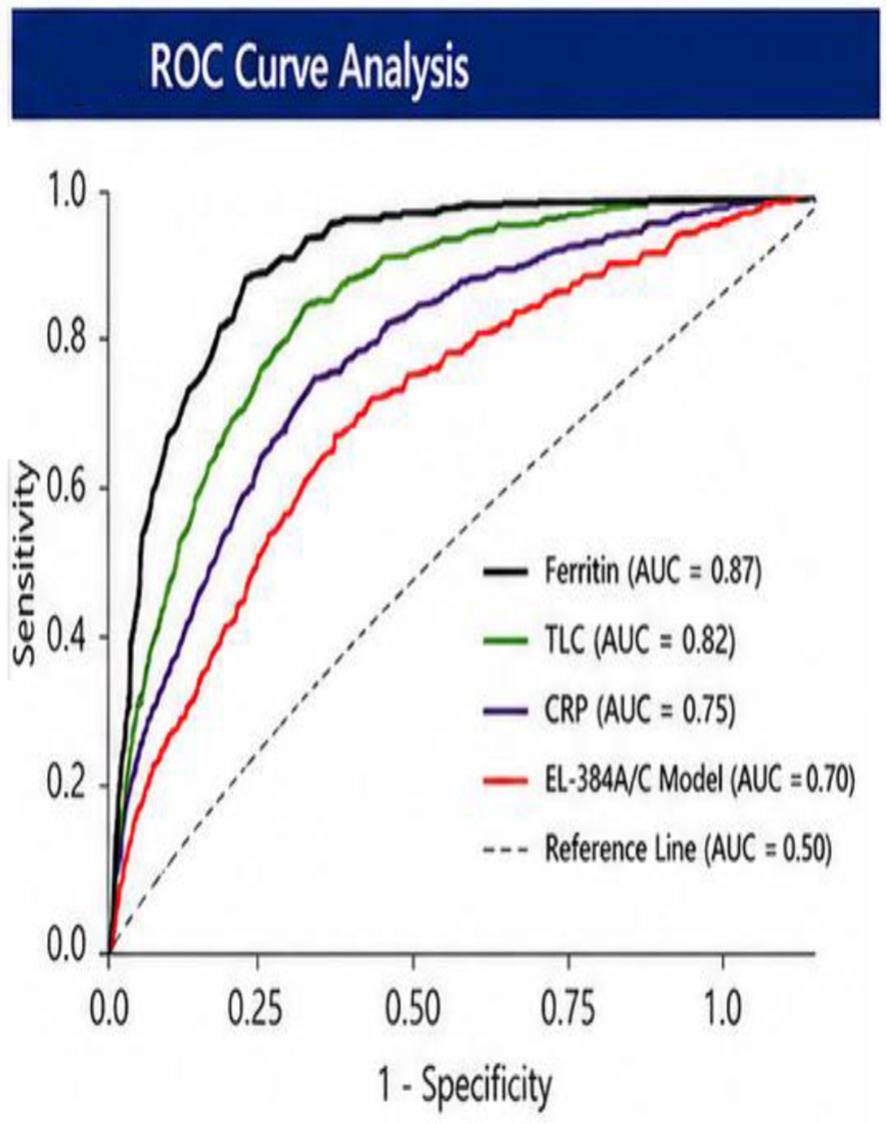
Receiver operating characteristic (ROC) curves evaluating the predictive performance of the EL-384A/C genotype and inflammatory biomarkers for COVID-19 severity. The curves compare the discriminatory performance of EL-384A/C, ferritin, CRP, and a combined model incorporating EL-384A/C and ferritin.

ROC analysis demonstrated fair discrimination for predicting elevated TLC using EL-384A/C alone (AUC = 0.62; P = 0.01; **Table 9**). Ferritin performed better (AUC = 0.71; P < 0.001), while CRP was poor. A combined model incorporating EL-384A/C and ferritin improved predictive performance (AUC = 0.75; P < 0.001; **Figure 4**; **Supplementary Tables 5**, **6**).

**Table 9:** ROC curve performance of EL-384A/C and clinical biomarkers for predicting elevated TLC.

Predictor	AUC (95% CI)	Sensitivity (%)	Specificity (%)	P-value
EL-384A/C (Genotype)	0.62 (0.54–0.70)	61.2	60.4	0.01
Ferritin	0.71 (0.64–0.79)	68.9	66.7	<0.001
CRP	0.59 (0.51–0.67)	57.1	58.3	0.08
Combined model (EL-384A/C + Ferritin)	0.75 (0.68–0.82)	72.4	70.1	<0.001

AUC, area under the ROC curve. Combined models were generated using logistic regression probabilities. P-values indicate AUC significantly different from 0.5.

### 3.10 Network and pathway enrichment analysis

**Figure 5:**
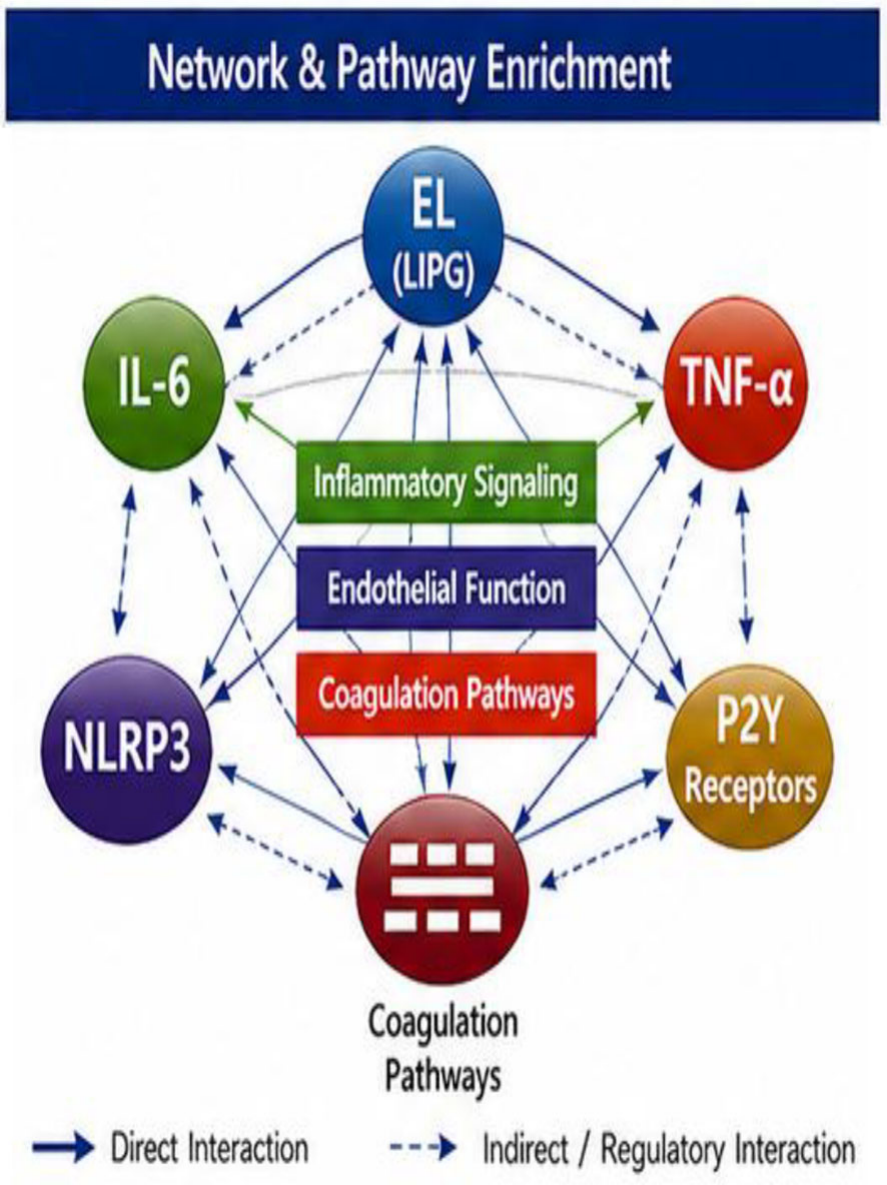
Network and pathway enrichment analysis illustrating interactions between endothelial lipase (LIPG) and key inflammatory mediators involved in COVID-19 pathogenesis. The network highlights connections with IL-1–centered inflammatory pathways, cytokine signaling (IL-6 and TNF-α), and endothelial dysfunction–related coagulation pathways.

Protein–protein interaction and pathway enrichment analyses indicated that EL-384A/C is associated with IL-1–centered inflammatory networks, involving IL-6, TNF-α, NLRP3, and coagulation pathways. These network-level insights provide mechanistic support for the observed clinical and biomarker associations, highlighting the role of EL-384A/C in modulating inflammatory and thrombotic responses in COVID-19 (**Figure 5**).

### 3.11 Integrated summary of findings

**Figure 6:**
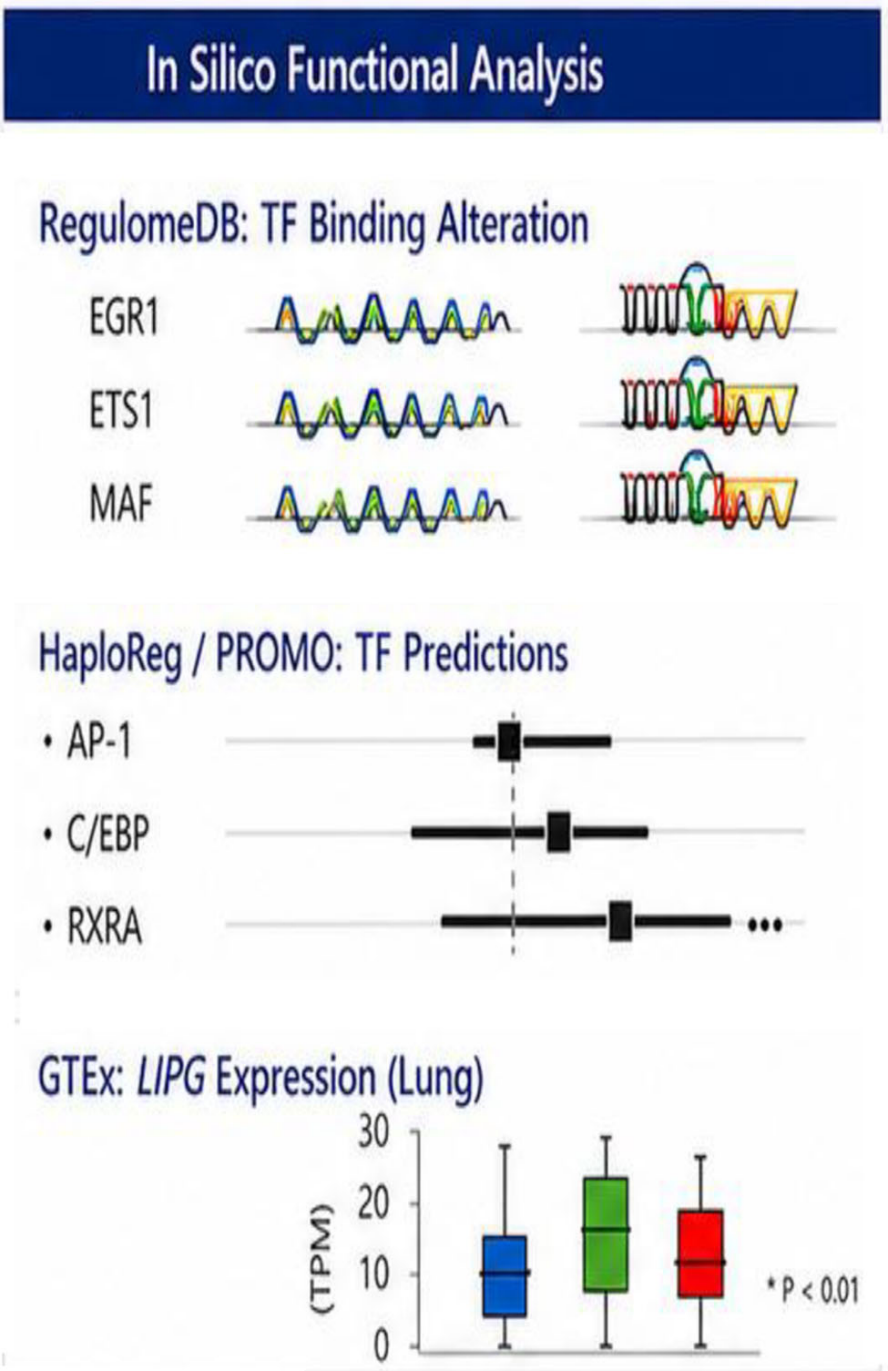
In-silico functional annotation of the LIPG promoter variant rs3813082. Regulatory annotation and transcription factor binding predictions indicate that the −384A/C substitution may alter motifs for inflammatory transcription factors, including NF-κB, SP1, and AP-1, supporting a potential regulatory role in immune-responsive pathways.

EL-384A/C does not influence COVID-19 susceptibility, but the C allele confers strong protection against disease severity with a clear dose-response trend. The variant shows selective association with hyperferritinemia and leukocytosis, supported by functional regulatory predictions, ROC analyses, and network-level pathway insights (**Figures 1**–**6**).

## 4 Discussion

In this study, we examined the LIPG −384A/C (rs3813082) promoter polymorphism in a well-characterized cohort of COVID-19 patients from Western India. While the variant did not influence overall susceptibility to SARS-CoV-2 infection, the C allele appeared to be protective against disease severity, showing a decreasing trend from mild to critical cases. Additionally, the AC genotype and C allele were associated with elevated ferritin and total leukocyte count (TLC), suggesting a potential modulatory role in host inflammatory responses.

Although the associations between the LIPG −384A/C polymorphism and inflammatory markers such as ferritin and total leukocyte count (TLC) were statistically significant, the discriminatory performance observed in the ROC analysis was modest (AUC = 0.62). Therefore, this genetic variant alone is unlikely to serve as a highly specific standalone biomarker for predicting COVID-19 severity. Instead, it may contribute to integrated risk prediction models that combine genetic variants with clinical and inflammatory biomarkers. Furthermore, future investigations incorporating more specific indicators of endothelial dysfunction, such as von Willebrand factor or angiopoietin-2, may provide deeper mechanistic insight into the role of endothelial pathways in COVID-19 pathophysiology.

Despite certain limitations, the present findings provide preliminary evidence supporting a potential role of the LIPG −384A/C promoter polymorphism in modulating host inflammatory responses associated with COVID-19 severity.

### 4.1 Host genetics and COVID-19 outcomes

COVID-19 exhibits substantial clinical heterogeneity, influenced by both viral and host factors. Host genetic variants have been shown to modulate immune responses and clinical outcomes. GWAS have identified loci associated with severity, including 3p21.31 (LZTFL1, SLC6A20) and other immune-related genes (36). Our findings that the EL-384C allele is associated with milder disease are consistent with the concept that regulatory variants can influence disease severity without affecting susceptibility. Variants in innate immune pathways, including TLRs and interferon signaling, similarly modulate host responses rather than viral entry (37).

### 4.2 Sex-specific genetic effects

We observed a higher frequency of the protective C allele in females, reflecting potential sex-dependent modulation of COVID-19 outcomes. These results align with epidemiological data showing higher severity and mortality in males, likely due to immunological and hormonal differences (36). Sex-linked genetic effects, such as TLR7 variants on the X chromosome, further support differential genetic contributions to disease severity (38).

### 4.3 Inflammatory biomarkers

Systemic inflammation is a hallmark of severe COVID-19. Our results showed that the AC genotype and C allele were enriched in patients with elevated ferritin and TLC, highlighting a selective influence on inflammatory responses. These findings are consistent with reports that regulatory variants in immune pathways modulate cytokine production and inflammatory cascades, affecting disease progression. The allele frequency of the EL-384A/C (rs3813082) polymorphism observed in our cohort is comparable to that reported in Asian populations, where the minor C allele frequency has been estimated to range between approximately 16–22%. This similarity suggests that our study population reflects the broader Asian genetic background. Such population-specific genetic variation may influence the strength of associations observed in genetic susceptibility studies.

### 4.4 Functional implications

*In silico* analyses indicated that rs3813082 resides in a regulatory promoter region of LIPG, potentially altering transcription factor binding (NF-κB, SP1, AP-1, and GATA). These factors play central roles in innate immune activation, and altered binding may attenuate pro-inflammatory signaling, thereby contributing to milder clinical phenotypes. Network and pathway analyses further demonstrated IL-1–centered inflammatory networks, supporting the functional relevance of EL-384A/C in modulating host responses (**Figure 5**).

Although these computational analyses provide valuable biological insights, they should be interpreted as predictive rather than direct functional evidence. Experimental validation using approaches such as luciferase reporter assays, chromatin immunoprecipitation (ChIP) to assess transcription factor binding, and measurement of endothelial lipase expression in relevant cellular systems would be required to confirm the regulatory effects of this polymorphism.

The conceptual model presented in **Figure 2** integrates these genetic associations with predicted regulatory mechanisms and previously reported endothelial inflammatory pathways, and therefore represents a hypothesis-generating framework that requires experimental validation.

### 4.5 Clinical relevance and predictive value

While a single variant has limited predictive utility, ROC analyses showed that EL-384A/C, especially when combined with ferritin, could better discriminate elevated TLC than conventional markers alone. These findings suggest potential value in integrating EL-384A/C into composite risk models for early identification of patients at risk of excessive inflammation.

### 4.6 Limitations and future directions

The present study has several limitations that should be acknowledged. First, the sample size was relatively modest, which may limit the statistical power to detect subtle genetic effects on COVID-19 susceptibility. Second, the functional implications of the LIPG −384A/C (rs3813082) polymorphism were inferred primarily from in silico analyses, and direct experimental validation—such as luciferase reporter assays, chromatin immunoprecipitation (ChIP), or expression studies—was not performed. Third, the findings were derived from a single regional cohort and therefore require replication in larger, independent, and ethnically diverse populations to confirm their generalizability.

Although individuals with major comorbid conditions were excluded during recruitment to minimize potential clinical confounding, it remains possible that other unmeasured host genetic, environmental, or immunological factors may have influenced disease severity. Therefore, residual confounding cannot be completely ruled out. Future studies integrating larger multi-center cohorts, broader clinical covariates, and functional genomic analyses will be important to clarify the independent contribution of the LIPG −384A/C polymorphism to COVID-19 outcomes and host inflammatory responses.

## 5 Conclusions

The EL-384A/C polymorphism does not affect overall susceptibility to SARS-CoV-2 infection but confers robust protection against COVID-19 severity. The C allele selectively influences inflammatory biomarkers, supported by functional and network analyses. These findings underscore the role of regulatory genetic variation in shaping host inflammatory responses and contribute to understanding the genetic determinants of COVID-19 clinical heterogeneity.

## Data Availability

The datasets presented in this study can be found in online repositories. The names of the repository/repositories and accession number(s) can be found in the article/**Supplementary Material**.
