# Supplementary material for: Immunogenetic modulation of endothelial inflammation by the LIPG −384A/C promoter variant influences COVID-19 severity
Source: Front Immunol. 2026 Jun 3;17:1785738. doi: 10.3389/fimmu.2026.1785738 (PMC13273450; doi:10.3389/fimmu.2026.1785738)

## **Supplementary Tables**

### ****Table S1. Association of EL-384A/C with Renal and Liver Function Parameters****

| **Biomarker** | **Comparison** | **OR (95% CI)** | **P-value** |
| --- | --- | --- | --- |
| Creatinine | Elevated vs Normal | 1.12 (0.55–2.28) | 0.76 |
| SGOT | Elevated vs Normal | 0.98 (0.49–1.95) | 0.95 |
| Bilirubin | Elevated vs Normal | 1.31 (0.60–2.88) | 0.49 |
| Blood urea | Elevated vs Normal | 0.89 (0.42–1.88) | 0.76 |

### ****Table S2. Expanded Inflammatory Marker Associations****

| **Biomarker** | **Variant** | **OR (95% CI)** | **P-value** |
| --- | --- | --- | --- |
| CRP | AC vs AA | 0.50 (0.23–1.03) | 0.07 |
| D-dimer | AC vs AA | 2.22 (0.40–13.10) | 0.42 |

### ****Table S3. Hardy–Weinberg Equilibrium and Genotyping Quality Control****

| **Group** | **χ²** | **P-value** | **Status** |
| --- | --- | --- | --- |
| Controls | 0.45 | 0.50 | In equilibrium |
| Cases | 1.92 | 0.17 | In equilibrium |

### ****Table S4. Full Logistic Regression Outputs****

| **Outcome** | **Model** | **OR** | **95% CI** | **P** |
| --- | --- | --- | --- | --- |
| Severity | Unadjusted | 4.15 | 2.10–8.22 | <0.001 |
| Severity | Age-adjusted | 3.88 | 1.94–7.76 | <0.001 |
| Severity | Age + Sex adjusted | 3.72 | 1.81–7.64 | <0.001 |

### ****Table S5. ROC Performance for Predicting Hyperferritinemia****

| **Predictor** | **AUC (95% CI)** | **P-value** |
| --- | --- | --- |
| EL-384A/C | 0.69 (0.61–0.77) | <0.001 |
| TLC | 0.66 (0.58–0.74) | 0.002 |
| Combined | 0.74 (0.67–0.81) | <0.001 |

### ****Table S6. ROC Performance for Predicting Elevated TLC****

| **Predictor** | **AUC (95% CI)** | **P-value** |
| --- | --- | --- |
| EL-384A/C | 0.62 (0.54–0.70) | 0.01 |
| Ferritin | 0.71 (0.64–0.79) | <0.001 |

### ****Table S7. In Silico Functional Annotation of LIPG rs3813082****

| **Tool** | **Evidence** |
| --- | --- |
| RegulomeDB | Score 2b – likely regulatory |
| HaploReg | Alters TF motifs (SP1, NF-κB) |
| JASPAR / PROMO | Differential transcription factor binding |
| GTEx | eQTL signal for LIPG expression |

**Supplementary Figure S1. Representative PCR-RFLP genotyping results of the EL-384A/C (rs3813082) polymorphism resolved on a 3% agarose gel.**
Lane M: DNA marker; Lane 2: AA genotype showing a single band at 254 bp; Lane 3: AC genotype showing 254 bp, 142 bp, and 112 bp fragments; Lane 4: CC genotype showing 142 bp and 112 bp fragments following restriction enzyme digestion.


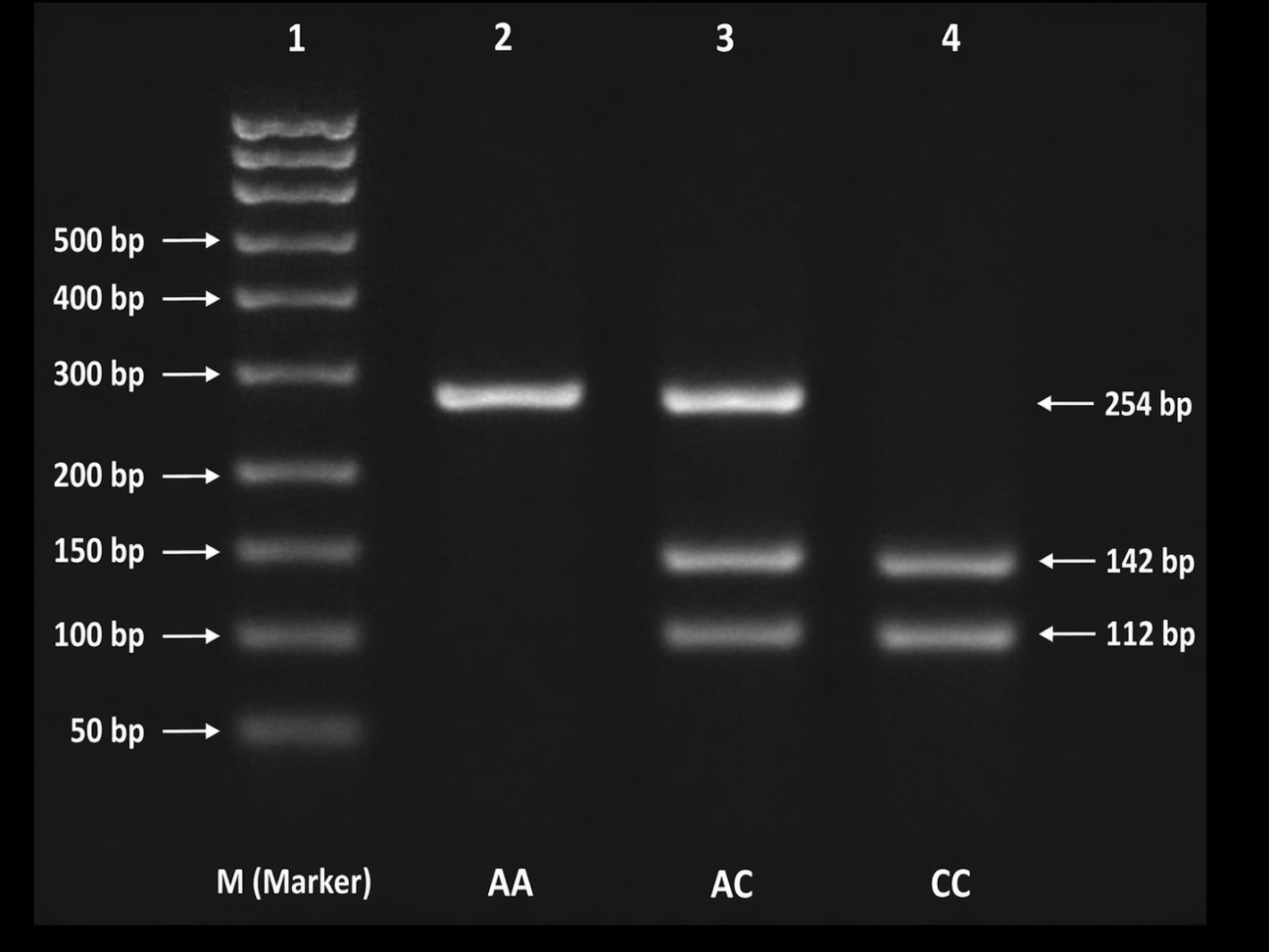

Supplement: Supplementary file 1 [file DataSheet1.docx]
